# Multi-Kingdom Fecal Microbiota Alterations in Horses with Severe Equine Asthma

**DOI:** 10.3390/microorganisms14020484

**Published:** 2026-02-17

**Authors:** Rafaela Santos, Laszlo Hunyadi, Emily Sundman, Luis Morales Luna, Sarah Cate Hyde, Makala Cain, Kagan Migl, Jacob Ancira, Craig Tipton, Fernanda Rosa

**Affiliations:** 1 School of Veterinary Medicine, Texas Tech University, Amarillo, TX 79106, USA; rafsanto@ttu.edu (R.S.); lhunyadi@ttu.edu (L.H.); esundman@ttu.edu (E.S.); l.morales@ttu.edu (L.M.L.); sarah-cate.hyde@ttu.edu (S.C.H.); makala.fussell@ttu.edu (M.C.); kmigl@ttu.edu (K.M.); 2 Department of Biological Sciences, Texas Tech University, Lubbock, TX 79409, USA; 3 RTL Genomics, MicroGenDX, Lubbock, TX 79413, USA

**Keywords:** allergies, asthma, equine, fecal microbiota, inflammation

## Abstract

Severe equine asthma (SEA) is a chronic inflammation of airways affecting ~14–20% of adult horses in the Northern Hemisphere. SEA is characterized by a mixed phenotype of T helper cell responses with marked neutrophilia in the bronchoalveolar lavage fluid (BALF) of affected horses. Human studies have demonstrated the impact of gut microbiota in many diseases, including asthma susceptibility and severity. However, the potential role of the gut–lung axis in the development and persistence of SEA remains to be determined. This study aimed to identify key bacterial, archaeal, and fungal microbiota alterations in the feces of horses with severe neutrophilic asthma (n = 4) compared to healthy horses (n = 8). Archaea alpha diversity was lower in the feces of SEA-affected horses, but with high abundance of archaea genus *Candidatus Nitrosocosmicus*, which impacts hydrogen metabolism in horses with SEA. Other key bacterial and fungi species differences lower in SEA included *Blautia* and *Alternaria*, respectively. *Blautia* is associated with positive metabolic health due to its fibrolytic capabilities. Overall, our findings indicate that horses experiencing severe neutrophilic asthma have an imbalance in the intestinal microbiota that may exacerbate systemic inflammatory responses through the gut–lung axis.

## 1. Introduction

Severe equine asthma (SEA) is a chronic respiratory illness of adult horses exposed to environmental aeroallergens. Multiple allergens have been shown to induce airway inflammation in SEA-affected horses including hay, fungi species, microbial toxins, forage mites, pollen, plant debris, and inorganic particles [1]. Clinically SEA is characterized by reversible bronchospasm, airway inflammation and mucus buildup, affecting about 20% of the adult horses in the Northern Hemisphere [2]. Different approaches can be used for diagnostic confirmation of SEA, including airway endoscopy, where excessive tracheobronchial mucus indicates severe disease rather than mild asthma [3]; airway differential cytology in bronchoalveolar lavage fluid (BALF) with neutrophilia (neutrophils ≥ 20–25% of total BALF cells) [4]; and pulmonary function testing, revealing airway hyperresponsiveness and constriction during exacerbation [5]. This complex disease varies in levels of susceptibility, inflammation phenotypes, and host-specific susceptibility to allergens at an individual level [6]. SEA is often compared to human asthma, which is typically characterized by increased levels of immunoglobulin E (IgE) associated with T helper cell type 2 (Th2) responses resulting in the influx of eosinophils into the airways [7]. However, SEA is typically characterized by a mixed phenotype involving increased T helper cell responses (Th1, Th2, and Th17) with a neutrophilic infiltration in the BALF of affected horses [5,8]. Several reports have shown different cytokine profiles (e.g., IL-1b, IL-8, IFN-g, TNF-a, IL-17) in SEA-affected horses with a Th1, Th2, Th17, or mixed response mediated by T helper cells [9,10,11]. Yet, the precise cytokine profile and the immunological mechanisms of the disease remain unclear.

In humans, microbial dysbiosis (an imbalance in microbial composition) in the gut and lungs during infancy is one of the risk factors associated with childhood asthma development through the gut–lung axis [12,13]. For instance, germ-free mice studies have demonstrated that mice with lower microbial diversity were more susceptible to developing T helper 2-mediated response to different types of allergens [14,15]. In different species, including humans and equine, several mediators produced by commensal microorganisms such as short-chain fatty acids (SCFAs) and peptidoglycans can immunologically support the tolerance upon allergen exposure by enhancing the function and development of regulatory T cells and maintaining a balanced response to potential allergens and other foreign substances [16,17]. In addition to early-life tolerance development, alterations in the gastrointestinal microbiota of older individuals may influence metabolite profiles in the gastrointestinal tract, thereby contributing to systemic and pulmonary inflammation [18]. Furthermore, several studies have shown distinct gut microbial profiles in humans with respiratory diseases compared to healthy individuals, highlighting the biological relationship between the gut and lungs [19]. Although the precise mechanisms underlying this phenomenon remain to be fully elucidated, growing evidence supports the existence of a bidirectional gut–lung axis through which intestinal and respiratory systems communicate via immune, microbial, and metabolic pathways [20]. Alterations in gut microbial composition and function can influence pulmonary immune responses by shaping systemic inflammation, mucosal immune priming, and the production of microbial-derived metabolites that circulate to the lungs [21]. Also, respiratory disease and inflammation can disrupt gut homeostasis, further amplifying immune dysregulation [22]. In summary, these interactions suggest that the gut–lung axis plays a critical role in both the etiology and progression of respiratory diseases including asthma and represents a promising target for novel preventive and therapeutic strategies aimed at modulating host–microbiota interactions to improve airway health. However, the underlying mechanisms between gut dysbiosis and severe neutrophilic asthma have yet to be described in the equine population.

As recently reviewed by Leduc et al. [23], studies showing the microbial dynamics in horses with asthma (e.g., allergic asthma or mild/moderate asthma) are very limited. Additionally, equine studies focusing on severe asthma or mild asthma have reported only bacterial alterations [24,25]. The equine gastrointestinal tract and respiratory systems harbor diverse microbial ecosystems encompassing not only bacteria but also archaea, fungi, viruses, and parasites. While bacterial components have received considerable attention, archaea and fungi remain understudied despite their potential significance in health and disease states.

To our knowledge, multi-kingdom fecal microbiota alterations in horses with severe neutrophilic asthma have not been described. Therefore, it was hypothesized that key gut microbial populations, which enhance the airway inflammatory response, are decreased in horses diagnosed with severe neutrophilic asthma compared to non-asthmatic horses. This study aimed to identify key microbial population differences including bacterial, archaeal, and fungal abundance and diversity in horses with severe neutrophilic asthma compared to clinically healthy horses. Hence, this study used fecal microbial profiling as a proxy for distal intestinal microbial dynamics in horses, as previously demonstrated by Costa et al. [26].

## 2. Materials and Methods

### 2.1. Study Design

This study used opportunistically collected samples obtained from animals enrolled in an independent, concurrently conducted research project that was approved by the Texas Tech University Institutional Animal Care and Use Committee (IACUC# 2023-1307). Sample collection was performed for the primary objectives of the parent study, and no additional procedures, interventions, or animal handling were required specifically for the purposes of the present investigation.

The demographic information of horses selected for this study is summarized in Table 1. Horses ranged in age from 5 to 20 years and were of mixed breed and gender (see Table 1 for detailed information for each animal). Based on the animals’ clinical history (survey based), horses used in this study were not on any specific asthma therapy or medication prior to or on the day of sample collections. On sampling days, each horse received a physical exam by a trained equine veterinarian and a BALF sample was collected for differential cytology analysis performed at the Texas A&M Veterinary Medical Diagnostic Laboratory (Canyon, TX, USA). Collection of BALF samples from each horse was performed according to standard procedures [27]. Briefly, horses were sedated with detomidine (0.005–0.01 mg/kg IV) and butorphanol (0.02–0.04 mg/kg IV). An equine BAL tube was inserted up the left or right nostril into the ventral and middle meatus into the pharynx, and the tube was passed into the trachea. Two hundred and fifty milliliters of sterile saline was administered through the BAL tube and then the fluid was aspirated back via the same syringe. The collected BALF was processed by cytocentrifugation, and a 400 differential cell count was performed by a board-certified clinical pathologist [28]. Diagnosis of asthma was determined by a board-certified internal medicine specialist and cellular phenotype was determined according to current recommendations [5].

### 2.2. Experimental Groups

Bronchoalveolar lavage fluid cytology was used to assign horses to one of the two groups: healthy or severe equine asthma (SEA). Criteria for BALF cytology classification was similar to that previously reported by Davis and Sheats et al. [29], which are in accordance with the consensus statement on inflammatory airway disease published by the American College of Veterinary Internal Medicine [5]. Horses were enrolled in the healthy group if BALF cytology had ≤6% neutrophils, ≤2% mast cells, and ≤1% eosinophils, along with no clinical signs of illness at the physical exam. SEA horses presented a BALF cytology of ≥20% neutrophils accompanied by clinical signs of respiratory distress (e.g., coughing and/or wheezes) at the physical exam. From the equine cohort evaluated (n = 70), a total of 8 horses were classified as healthy while 4 horses were diagnosed with neutrophilic SEA. Horses with mild to moderate neutrophilic, eosinophilic, or mastocytic asthma were excluded from the present study. The BALF inflammatory phenotype for each horse used in this study is presented in Table 2.

### 2.3. Sample Collection and DNA Extraction

Rectal swabs (Cat# 14-907-20, Fisher Scientific Inc., Waltham, MA, USA) were collected from all horses immediately after BALF sample collection. Rectal swabs were placed in dry ice until transportation to the Texas Tech School of Veterinary Medicine (samples collected in HI and VA were shipped overnight in dry ice), and samples were subsequently stored at −80 °C until DNA extraction. Genomic DNA was extracted from all rectal swabs using a QIAamp^®^ Power-fecal^®^ Pro DNA kit (Cat# 51804, Qiagen, Hilden, Germany) according to the manufacturer’s instructions with few modifications. Briefly, the extraction protocol was modified to include a mechanical lysis step in which swabs were immersed in 1 mL of lysis solution in tubes containing beads (provided in the kit) and homogenized using a bead beater homogenizer (Bead Blaster 24R, model D2400-R, Benchmark, Tempe, AZ, USA) at 3000 RPM, 1 min per cycle for a total of 10 cycles at room temperature. Quantification and quality of isolated DNA were assessed using a Nanodrop™ spectrophotometer (Nanodrop Onec, Thermo Fisher Scientific, Madison, WI, USA).

### 2.4. Targeted Bacterial, Fungal, and Archaeal Amplicon Sequencing

Samples were amplified for sequencing in a two-step process, similar to previous work [30]. Forward primers were constructed (5′–3′) by adding forward Illumina overhang adapters (TCGTCGGCAGCGTCAGATGTGTATAAGAGACAG) to respective bacterial, fungal, and archaeal forward primers (listed in Table 3) and reverse primers by adding reverse Illumina overhang adapters (GTCTCGTGGGCTCGGAGATGTGTATAAGAGACAG) to respective reverse primers (Table 3). For the targeted bacterial profiling the V1–V3 hypervariable regions of the 16S rRNA gene were selected for amplicon sequencing based on previous studies that have demonstrated that the V1–V3 region provides strong, often species-level, discrimination for many bacterial types with a broader phylogenetic resolution [31,32,33,34].

Amplifications were performed in 20 µL reactions with Qiagen AllTaq master mix (Qiagen Inc, Valencia, CA, USA), 1 µL of each 5 µM primer, and 1 µL of template. Reactions were performed on ABI Veriti thermocyclers (Applied Biosytems, Carlsbad, CA, USA) under the following thermal profile: 95 °C for 2 min, then 35 cycles of 95 °C for 5 s, 55 °C for 15 s, 72 °C for 10 s, followed by one cycle of 72 °C for 10 min and 4 °C hold.

Products from the first-stage amplification were added to a second PCR based on qualitatively determined concentrations. Primers for the second PCR were designed based on the Illumina Nextera PCR primers (F: AATGATACGGCGACCACCGAGATCTACAC[i5index]TCGTCGGCAGCGTC; R: CAAGCAGAAGACGGCATACGAGAT[i7index]GTCTCGTGGGCTCGG). The second-stage amplification repeated the previous thermal conditions for an additional 8 cycles. Amplification products were visualized with eGels (Life Technologies, Grand Island, NY, USA). Products were then pooled equimolarly and each pool was size selected in two rounds using SPRIselect Reagent (BeckmanCoulter, Indianapolis, IN, USA) in a 0.80 ratio for both rounds. Size-selected pools were then quantified using the Qubit 4 Fluorometer (Life Technologies) and loaded on an Illumina MiSeq (Illumina, Inc. San Diego, CA, USA) 2 × 300 flow cell at 10 pM.

### 2.5. Bioinformatic Processing

Bioinformatic processing and quality filtering generally followed previous work [35]. For bacterial and archaeal data, denoising of sequence reads and chimera detection were performed using Usearch11 [36] and PEAR [37] was used for paired read stitching. Zero-radius OTUs (zOTUs) were identified using UNOISE2 [38]. Fungal data were processed using a previously used method for 97% OTU clustering [35] as the more stringent zOTU method was observed to have greater dropout for fungal reads. Taxonomic assignment of the representative sequences was performed using SINTAX [39] and the RTL Genomics in-house taxonomic reference database, which is adapted from the National Center for Biotechnology Information (NCBI) database with additional curation. Multiple sequence alignment was performed for downstream analysis using MUSCLE [40] for fungal OTUs or SSU-ALIGN [41] for bacterial and archaeal zOTUs. Phylogenetic tree estimation of representative sequences was performed using FastTree2 [42] for all assays.

### 2.6. Statistical Analysis

The statistical analyses for the microbiota composition datasets were conducted in R version 4.1.0. using the vegan [43] and phyloseq [44] packages. First, alpha diversity was examined using overall richness, Shannon diversity, Hill1 diversity, and Hill1 phylogenetic diversity [45]. A two-tailed t-test was used to compare diversity metrics between healthy and SEA groups. ANCOM-BC was used to identify differentially abundant taxa between each sample group (healthy and SEA). Here, a prevalence threshold of 0.8 (taxa in less than 20% of samples were excluded) with no adjustment applied to the *p*-value, to screen for potentially differentially abundant organisms. Associations identified by ANCOM-BC were further evaluated by a permutational approach that iteratively compared sets of 3 healthy versus 3 SEA animals to consider which species associations were the most robust. Among healthy and SEA groups, all possible combinations of 3 subjects were made, which resulted in 56 healthy subject combinations and 4 SEA subject combinations. For each of the individual taxa identified as differentially abundant, the mean relative abundance was calculated for the 3 subjects of every combination. Unweighted UniFrac matrices [46] were used to assess overall compositional differences between SEA and healthy animals (i.e., beta diversity). Principal coordinates analysis (PCoA) was performed to visualize overall sample clustering patterns based on differences in taxa. For quantitative testing, permutational multivariate analysis of variance (PERMANOVA) was used to formally test whether there were overall community differences between each group. Significance was declared at *p* ≤ 0.05. Estimates are shown as mean ± standard error of the mean.

## 3. Results

### 3.1. Fecal Microbiota Diversity

**Figure 1:**
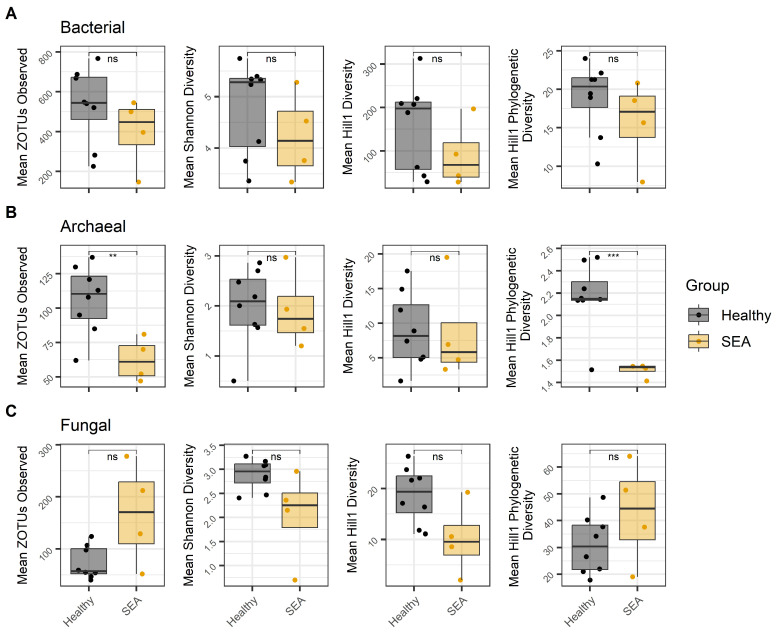
Alpha diversity matrices: estimated richness (ZOTUs = zero-radius operational taxonomic units), Shannon diversity index, Hill1 diversity index, and Hill1 phylogenetic index observed in fecal samples of healthy horses (n = 8) compared to horses with severe equine asthma (SEA; n = 4). The median value and first and third quartiles in each group are illustrated within each boxplot for: (**A**) Bacterial; (**B**) Archaeal; (**C**) Fungal. ns = non-significant; Significance was declared at *p* ≤ 0.05. *** *p* < 0.001; ** *p* = 0.01.

Within the alpha diversity measurements, no statistical differences were observed for the bacterial mean zOTUs, Shannon diversity index, Hill1, or Hill1 phylogenetic bacterial diversity indexes between healthy horses and the SEA group (Figure 1A).

Healthy horses had higher archaeal mean zOTUs observed compared to SEA horses (*p* = 0.01; Figure 1B). Similarly, a greater Hill1 phylogenetic diversity index was observed for the archaeal composition in the feces of healthy horses compared to SEA horses (*p* < 0.001; Figure 1B). However, no differences were observed in the Shannon or Hill1 diversity indexes within the archaeal profile.

No statistical differences were observed for the fungal OTUs, Shannon diversity, Hill1, or Hill1 phylogenetic bacterial diversity indexes between healthy horses and the SEA group (Figure 1C).

### 3.2. Fecal Bacterial Composition of Healthy and SEA-Affected Horses

**Figure 2:**
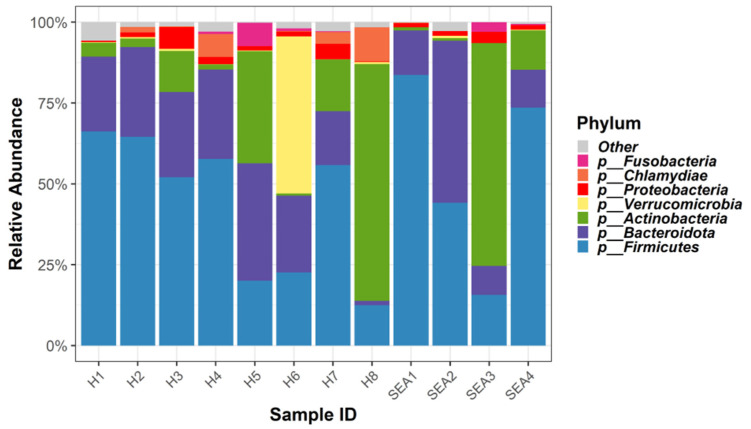
Relative abundance of the top 7 bacterial phyla in healthy (H) horses compared to horses with severe equine asthma (SEA). Individual abundances for each animal per group are presented.

Relative abundance of the top seven bacterial phyla in this equine cohort is presented in Figure 2.

**Figure 3:**
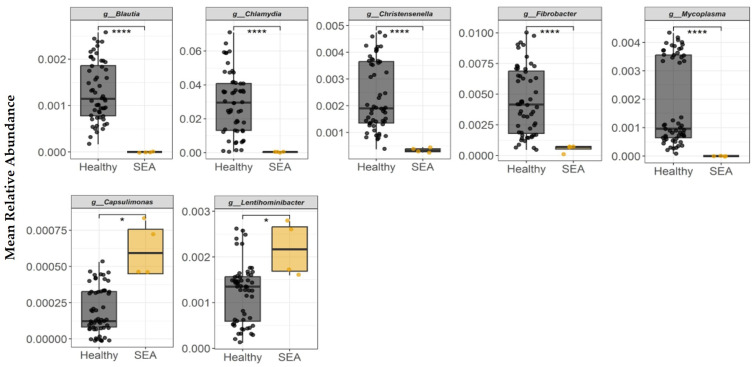
Mean relative abundance of bacterial genera identified in equine fecal samples to be differentially abundant between healthy and horses with severe equine asthma (SEA). Among both healthy and SEA groups, mean relative abundances were calculated for every possible combination of three SEA horses and three healthy horses, which resulted in 56 healthy subject combinations and 4 SEA subject combinations, and the mean relative abundance per group was calculated (*x*-axis). The median value and first and third quartiles in each group are presented in each boxplot. Significance was declared at * *p* ≤ 0.05. **** *p* < 0.0001.

Differentially abundant taxa between SEA and healthy horses were quantified by a two-step approach. First, the ANCOM-BC procedure was used to identify taxa that were non-randomly distributed between each group (*p* < 0.05). Second, mean relative abundances were calculated for every possible combination of three SEA horses and three healthy horses. Permuted mean relative abundances of each bacterial genera identified in fecal samples to be differentially abundant between groups are presented in Figure 3. *Blautia*, *Chlamydia*, *Christensenella*, *Fibrobacter*, and *Mycoplasma* relative abundances were higher in the feces of healthy horses compared to SEA horses (*p* < 0.0001). SEA horses had higher abundance of *Capsulimonas* and *Lentihominibacter* relative to healthy horses (*p* ≤ 0.04).

**Figure 4:**
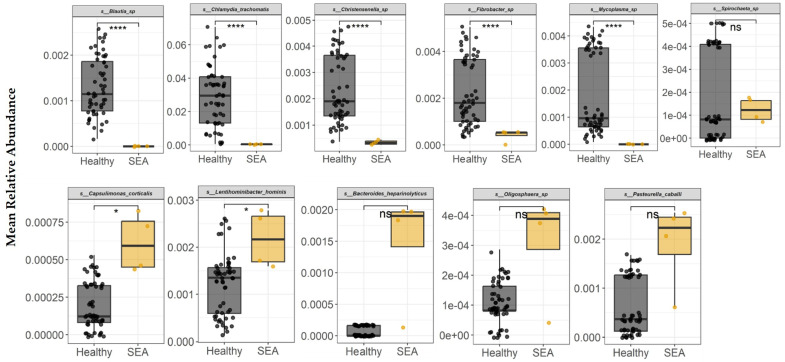
Mean relative abundance of bacterial species identified in equine fecal samples to be differentially abundant between healthy horses and horses with severe equine asthma (SEA). Among both healthy and SEA groups, mean relative abundances were calculated for every possible combination of three SEA horses and three healthy horses, which resulted in 56 healthy subject combinations and 4 SEA subject combinations, and the mean relative abundance per group was calculated (*x*-axis). The median value, first and third quartiles in each group are presented in each boxplot. ns = non-significant; Significance was declared at * *p* ≤ 0.05. **** *p* < 0.0001.

The differential abundance analysis was repeated at the species level, which identified seven species with significantly differing distributions across all permutations (Figure 4). At the bacterial species taxonomic level, healthy horses had higher abundance of *Blautia* sp., *Chlamydia trachomatis*, *Christensenella* sp., *Fibrobacter* sp., and *Mycoplasma* sp. in their feces compared to SEA horses (*p* < 0.0001), while *Capsulimonas corticalis* and *Lentihominibacter hominis* relative abundance was higher in SEA horses compared to healthy horses (*p* ≤ 0.04). *Bacteroides heparinolyticus*, *Oligosphaera* sp., *Pasteurella caballi*, and *Spirochaeta* sp. did not statistically differ between groups.

### 3.3. Fecal Archaeal Profile of Healthy and SEA-Affected Horses

**Figure 5:**
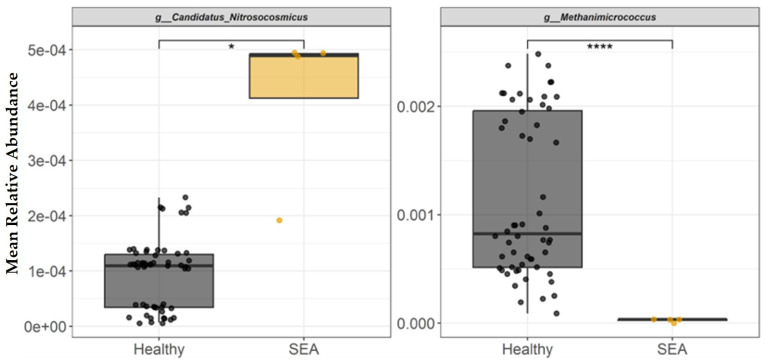
Mean relative abundance of archaeal genera identified in equine fecal samples to be differentially abundant between healthy horses and horses with severe equine asthma (SEA). Among both healthy and SEA groups, mean relative abundances were calculated for every possible combination of three SEA horses and three healthy horses, which resulted in 56 healthy subject combinations and 4 SEA subject combinations, and the mean relative abundance per group was calculated (*x*-axis). The median value and first and third quartiles in each group are presented in each boxplot. Significance was declared at * *p* ≤ 0.05. **** *p* < 0.0001.

The mean relative abundance of each archaeal genera identified in fecal samples to be differentially abundant between healthy and SEA horses is presented in Figure 5. *Methanimicrococcus* genus abundance was significantly decreased in the feces of SEA horses compared to healthy horses (*p* < 0.0001). In contrast, the fecal relative abundance of genus *Candidatus Nitrosocosmicus* was higher in the SEA group than in the healthy group (*p* = 0.02).

**Figure 6:**
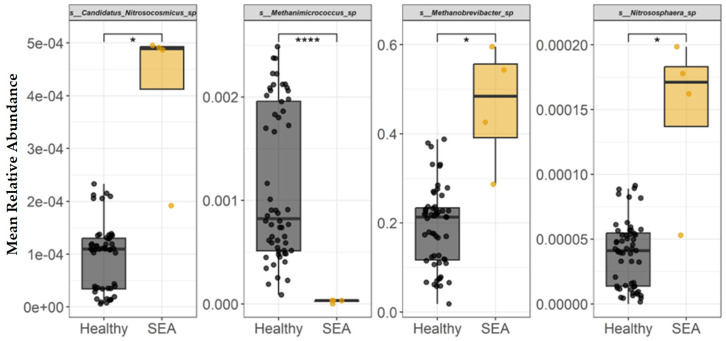
Mean relative abundance of archaeal species identified in equine fecal samples to be differentially abundant between healthy and horses with severe equine asthma (SEA). Among both healthy and SEA groups, mean relative abundances were calculated for every possible combination of three SEA horses and three healthy horses, which resulted in 56 healthy subject combinations and 4 SEA subject combinations, and the mean relative abundance per group was calculated (*x*-axis). The median value and first and third quartiles in each group are presented in each boxplot. Significance was declared at * *p* ≤ 0.05. **** *p* < 0.0001.

The mean relative abundance of each archaeal species identified in fecal samples to be differentially abundant between healthy and SEA horses is presented in Figure 6. At the archaeal species level, SEA horses had decreased abundance of *Methanimicrococcus* sp. compared to healthy horses (*p* < 0.0001). In contrast, SEA horses had higher abundance of *Candidatus Nitrosocosmicus* sp., *Methanobrevibacter* sp., and *Nitrososphaera* sp. compared to the fecal composition of healthy horses (*p* ≤ 0.04).

**Figure 7:**
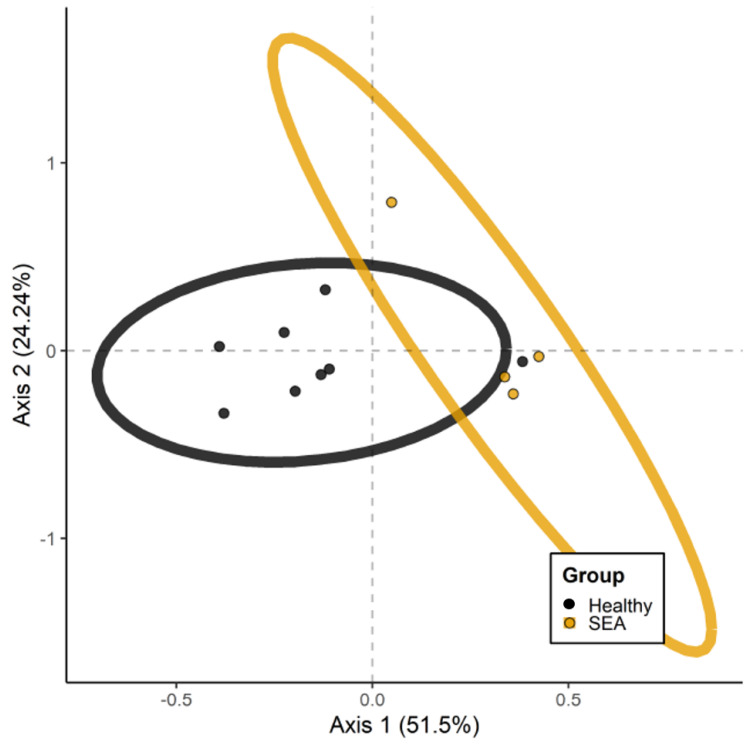
Principal coordinate analysis based on unweighted Unifrac distances for distinct archaeal taxa in fecal samples of healthy horses (n = 8) and horses with severe equine asthma (SEA; n = 4).

PERMANOVA performed on unweighted UniFrac distances revealed phylogenetically distinct archaea taxa alterations between healthy and SEA groups (*p* = 0.01; Figure 7).

### 3.4. Fecal Fungal Composition of Healthy and SEA-Affected Horses

**Figure 8:**
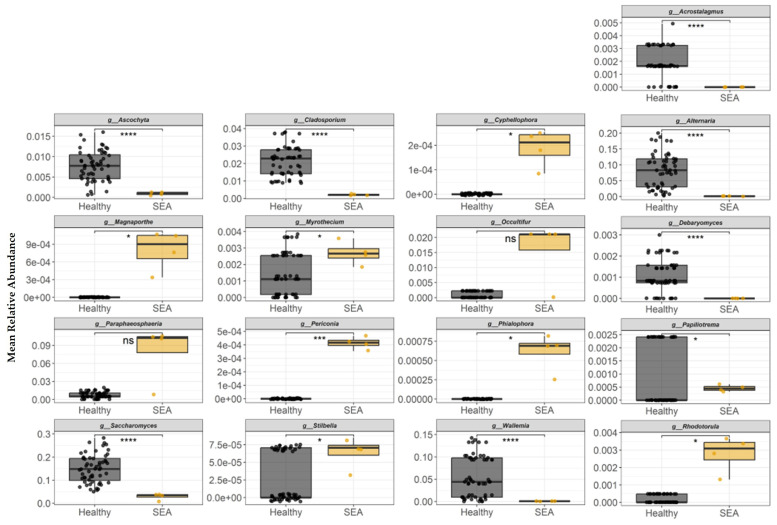
Mean relative abundance of fungal genera identified in equine fecal samples to be differentially abundant between healthy and horses with severe equine asthma (SEA). Among both healthy and SEA groups, mean relative abundances were calculated for every possible combination of three SEA horses and three healthy horses, which resulted in 56 healthy subject combinations and 4 SEA subject combinations, and the mean relative abundance per group was calculated (*x*-axis). The median value and first and third quartiles in each group are presented in each boxplot. Significance was declared at * *p* ≤ 0.05. **** *p* ≤ 0.01.

The mean relative abundance of fungal genera identified in fecal samples to be differentially abundant between healthy and SEA horses is presented in Figure 8. *Acrostalagmus*, *Alternaria*, *Ascochyta*, *Cladosporium*, *Debaryomyces*, *Papiliotrema*, *Saccharomyces*, and *Wallemia* fungi genera abundances were higher in the feces of healthy horses compared to SEA horses (*p* ≤ 0.01). In contrast, the fecal relative abundance of fungi genera *Cyphellophora*, *Magnaporthe*, *Myrothecium*, *Periconia*, *Phialophora*, *Rhodotorula*, and *Stilbella* were higher in the SEA group than in the healthy group (*p* ≤ 0.04).

**Figure 9:**
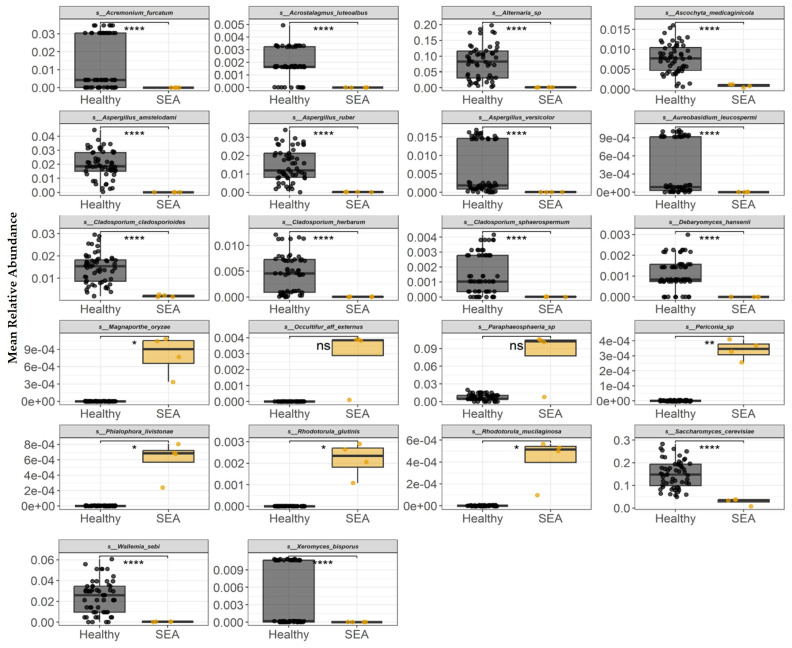
Mean relative abundance of fungal species identified in equine fecal samples to be differentially abundant between healthy and horses with severe equine asthma (SEA). Among both healthy and SEA groups, mean relative abundances were calculated for every possible combination of three SEA horses and three healthy horses, which resulted in 56 healthy subject combinations and 4 SEA subject combinations, and the mean relative abundance per group was calculated (*x*-axis). The median value and first and third quartiles in each group are presented in each boxplot. ns = non-significant; Significance was declared at * *p* ≤ 0.05. ** *p* ˂ 0.01. **** *p* < 0.0001.

The mean relative abundance of each fungi species identified to be differentially abundant between groups (modeled as a function of group) is presented in Figure 9. At the fungal species level, healthy horses had higher abundance of *Acremonium furcatum*, *Acrostalagmus luteoalbus*, *Alternaria* sp., *Ascochyta medicaginicola*, *Aspergillus amstelodami*, *Aspergillus ruber*, *Aspergillus versicolor*, *Aureobasidium leucospermi*, *Cladosporium cladosporioides*, *Cladosporium herbarum*, *Cladosporium sphaerospermum*, *Debaryomyces hansenii*, *Saccharomyces cerevisiae*, *Wallemia sebi*, and *Xeromyces bisporus* compared to the horses in the SEA group (*p* < 0.0001). In contrast, fungi species including *Magnaporthe oryzae*, *Periconia* sp., *Phialophora livistonae*, *Rhodotorula glutinis*, and *Rhodotorula mucilaginosa* were higher in the SEA group compared to healthy horses (*p* ≤ 0.03).

PERMANOVA performed on unweighted UniFrac distances revealed phylogenetically distinct fungal taxa alterations between healthy and SEA groups (*p* = 0.05; Figure S1).

## 4. Discussion

### 4.1. Overview

In equine medicine, SEA represents a chronic inflammatory airway disease characterized by neutrophilic inflammation, mucus hypersecretion, and reversible bronchoconstriction [5]. Several studies in humans and animals including horses indicate that gastrointestinal microbiota can be influenced by multiple factors including diet, systemic inflammation, and environmental conditions, and may in turn modulate allergic and inflammatory responses in distant organs such as the lungs [19,22,23]. The concept of the gut–lung axis proposes that intestinal dysbiosis (an imbalance in the gut microbial ecosystem) can exacerbate respiratory inflammation through altered production of immunomodulatory metabolites, impairment of the intestinal barrier function, and dysregulated immune responses [20,47]. Despite growing recognition of this axis in human and rodent asthma models, the characterization of fecal microbiota in horses with severe asthma remains limited, with significant knowledge gaps regarding specific microbial signatures, functional consequences, and therapeutic potential. Specific comparative data directly contrasting the gut microbiota composition of healthy horses with those suffering specifically from severe neutrophilic asthma remain limited in the published literature, representing a significant research gap that merits focused investigation.

### 4.2. Equine Gut–Lung Microbiota Alterations During Inflammation

Despite high individual variability, a “core microbiota” of key microorganisms have been suggested for equine species. Firmicutes and Bacteroidetes represent the largest phyla of bacterial communities across different gut compartments in healthy horses, which constitute 40–90%, including classes such as *Clostridia* and *Bacilli*, with additional contributions from Verrucomicrobia, Proteobacteria, Spirochaetes, and Actinobacteria [26,48]. These findings are aligned with our findings in which the most abundant phylum was Firmicutes for both healthy and SEA groups (43.5% in healthy and 56% in SEA), followed by Bacteroidota comprising 25% in healthy and 30% in SEA. *Clostridiales*, such as *Lachnospiraceae*, have been reported as part of the core microbiota in all mammals, due to their ability to produce butyrate, which provides protection to colonocytes [49]. Additionally, Bacteroidota are key degraders of complex polysaccharides and contribute to short-chain fatty acid (SCFA) production, thereby supporting gut homeostasis [50].

Following Firmicutes and Bacteroidetes, Proteobacteria phylum have been reported throughout the intestines and feces of healthy horses (~1–4%). Studies have shown Proteobacteria enriched in the upper gastrointestinal tract of healthy horses, predominantly in the ileum [26,51]. Some bacteria of the Proteobacteria phyla are known for their contribution to intestinal nitrogen fixation. However, overgrowth of Proteobacteria has been associated with intestinal inflammation and dysbiosis, including colic in horses [52,53]. In the present study, Proteobacteria accounted for less than 5% of the fecal microbiota in both healthy and SEA horses. This low abundance is consistent with previous reports showing that Proteobacteria, although part of the equine gut microbiota, are not dominant in the hindgut where fiber-fermenting phyla such as Firmicutes and Bacteroidota predominate [54]. Therefore, their low abundance in both groups likely reflects a stable hindgut environment, while the modest increase observed in SEA horses could indicate a subtle dysbiosis shift associated with airway disease and systemic inflammation.

Verrucomicrobia is another phylum reported in the equine cecum, small colon, rectum, and feces, but with conflicting results regarding its relative abundance [26,53,55]. In the present study, Verrucomicrobia was present in healthy horses, with 10% relative abundance, while 1% was detected in the feces of horses with SEA. Akkermansia, a mucin-degrading genus of Verrucomicrobia phyla, also supports mucosal integrity and reduces inflammation [56]. Thereby, low abundance of Verrucomicrobia bacteria in domesticated horses may contribute to their heightened susceptibility to gastrointestinal inflammation [57]. In accordance with these previous studies, our findings demonstrated that at the phylum level, Firmicutes, Bacteroidota, Actinobacteria, and Verrucomicrobia accounted for >90% of relative abundance in our equine cohort. At the species level, *Fibrobacter*, *Christensenella*, and *Blautia*, which are bacterial species often associated with commensal protective function [58], were increased in the healthy group compared to SEA horses.

Pioneering work by Leclère and Costa [25] provided the first comprehensive characterization of fecal microbiota in horses with asthma across different environmental and dietary conditions. They reported that asthmatic horses (experiencing exacerbation) had lower bacterial abundance compared to healthy horses, which was affected by changes in diet and environment (e.g., pasture, good hay, dusty hay). For instance, asthmatic horses eating poor-quality hay had a significant decrease in *Fibrobacter* compared to healthy horses on a hay diet [25]. This heightened susceptibility to environmental perturbations suggests that the gut microbiota of asthmatic horses exists in a more fragile equilibrium, with reduced resilience to external stressors. The inability of asthmatic horses to appropriately upregulate fibrolytic bacteria like *Fibrobacter* in response to hay-based diets may compromise their capacity to generate adequate SCFAs, potentially perpetuating systemic inflammation. It has been demonstrated that the *Fibrobacter* family is essential to maintain gut homeostasis by fermenting fibers and producing SCFAs, which have anti-inflammatory and immune-modulating properties [17]. Overall, these findings are in accordance with our study, where *Fibrobacter* sp. abundance was higher in the healthy group compared to SEA horses. Comparative studies of gut microbiota in horses with different gastrointestinal diseases provide additional context for understanding dysbiosis patterns. Park et al. [59] examined fecal microbiota in horses with large and small intestinal diseases compared to healthy controls, demonstrating that sick horses had significantly reduced species richness and bacterial diversity. Sick horses showed overgrowth of lactic acid bacteria families *Lachnospiraceae* and *Lactobacillaceae*, which are signs of gut dysbiosis and indicate an over-acidic environment (low pH) in the hindgut. This imbalance interferes with normal digestion and can lead to large intestinal colic and potentially laminitis [60].

The lungs harbor a distinct microbiota profile that plays a key role in regulating immunity, maintaining homeostasis, and protecting against respiratory pathogens [61]. Dysbiosis of the respiratory microbiome has been linked to persistent airway inflammation, as shown in both human and animal models [62,63]. Similarly, the intestinal microbiota is fundamental in shaping systemic immunity through a bidirectional crosstalk between the gut and lungs. This interaction is mediated by microbiota-derived metabolites, such as short-chain fatty acids, and can be influenced by diet, environment, disease, or medical interventions [23,64]. While most established mechanisms act from the gut to the lungs, alterations in microbial composition at either site may contribute to immune dysregulation and the development or persistence of airway diseases, including asthma [65]. In a previous equine study, pulmonary, oral, and nasal microbiota were similar within environmental conditions including horses on pasture, housed indoors receiving good-quality hay, or housed indoors receiving poor-quality hay. However, the microbiota of the lungs differed between horses with and without asthma during the period when airway inflammation was present in asthmatic horses [65]. Similarly to the fecal/gut microbiota described previously, the most abundant phylum in BALF samples of healthy and asthmatic horses reported by Fillion-Bertrand et al. [65] were Proteobacteria, followed by Bacteroidetes and Firmicutes. Such findings in the equine BALF bacterial profile align with other reports that demonstrated higher abundance of Proteobacteria microorganisms in horses with diarrhea [66] and in horses with colic [67] compared to healthy horses. Within this scenario, the shock organs of the horse are considered the respiratory tract and intestine. Therefore, many of the clinical signs of systemic anaphylaxis relate to these organs, including tachypnea, coughing, respiratory distress, colic, and diarrhea. Anaphylactic events are acute, generalized reactions, mediated via IgE and antigen binding, and secondary release of mast cell and basophil mediators [68]. Hence, the gut–lung axis communication results in a combination of respiratory distress and digestive failure simultaneously, which may be mediated by inflammatory mediators released into the circulation during gut inflammation affecting other organs, particularly the lungs [69]. Additionally, inter-individual heterogeneity in disease manifestation, despite standardized external conditions, suggests the involvement of intrinsic biological factors that modulate individual susceptibility and inflammatory response intensity. Gut microbiota alterations can potentially serve as mediators of equine asthma heterogeneity, as accumulating evidence demonstrates that microbial composition and function can profoundly influence systemic and mucosal immune responses, particularly through the gut–lung axis [70]. However, the cellular and molecular mechanisms underlying how airway obstruction and inflammation influence intestinal microbiota, and conversely how gut microbiota may modulate systemic inflammation in asthmatic horses, remain areas requiring further investigation.

### 4.3. Functional Characteristics of Gut Dysbiosis in Severe Equine Asthma

Severe equine asthma is characteristically marked by neutrophilic airway inflammation, with neutrophils playing both protective and pathogenic roles in disease pathogenesis [5]. The impact of gut microbiota on neutrophilic responses represents a critical mechanistic link in the gut–lung axis. Evidence from multiple species demonstrates that gut dysbiosis can promote neutrophil migration, activation, and extracellular trap (NET) formation through altered production of immunomodulatory metabolites and increased systemic inflammation [71]. Within this scenario, neutrophilic asthma represents a distinct inflammatory endotype characterized by predominant airway neutrophilia with a cytokine milieu driven largely by IL-17 family responses including IL-17A and IL-17F [72]. These cytokines, produced primarily by Th17 cells as well as innate lymphoid cells and γδ T cells, promote neutrophil recruitment and activation through the induction of chemokines (e.g., CXCL1, CXCL8) and granulopoietic factors such as granulocyte colony-stimulating factor (G-CSF) [73]. IL-17 signaling also enhances airway epithelial and stromal cell activation, contributing to mucus hypersecretion, airway remodeling, and sustained inflammation [74]. Overall, IL-17-mediated pathways link dysregulated innate and adaptive immunity to the persistence and severity of neutrophilic asthma, highlighting this axis as a critical driver of disease pathogenesis in asthmatic patients [75]. The IL-17 signaling pathway has emerged as a key immunological mediator connecting gut dysbiosis to neutrophilic airway inflammation [76]. In vitro studies using human peripheral blood mononuclear cells isolated from steroid-resistant asthmatic patients have shown that gut microbiota alterations are associated with elevated levels of inflammatory cytokines including IFN-γ and IL-17A [77]. Additionally, the gut microbiota can modulate Th17/Treg balance, with dysbiosis typically skewing toward pro-inflammatory Th17 responses at the expense of immunoregulatory Treg cells [78].

Human studies have consistently demonstrated that individuals with asthma, particularly those with severe or neutrophilic phenotypes, exhibit distinct gut microbiota profiles characterized by reduced α-diversity and altered taxonomic composition [79]. Experimental murine studies using house dust mite (HDM)-induced asthma models have shown that gut dysbiosis, characterized by enrichment of Proteobacteria and depletion of beneficial taxa, exacerbates allergic airway inflammation [80]. Particularly relevant to equine asthma are studies demonstrating the role of SCFA-producing bacteria in modulating neutrophilic inflammation. Human research has identified that depletion of genera such as Faecalibacterium, Bacteroides, and Ruminococcus in asthmatic individuals correlates with reduced fecal SCFA levels and increased airway inflammation [81]. The genus Faecalibacterium, a prominent butyrate producer, has been shown to correlate inversely with inflammatory markers in various inflammatory conditions [82], highlighting its potential as a beneficial commensal with broad anti-inflammatory properties. SCFAs exert immunomodulatory effects through multiple mechanisms relevant to asthma pathogenesis. They activate G-protein coupled receptors (GPR41 and GPR43) on immune cells, influencing chemotaxis, cytokine production, and cell differentiation [83]. Additionally, SCFAs, primarily butyrate, propionate, and acetate, function as histone deacetylase (HDAC) inhibitors, thereby altering gene expression patterns in immune cells to favor anti-inflammatory and regulatory phenotypes [84]. In the context of asthma, SCFA-mediated HDAC inhibition can enhance regulatory T cell (Treg) differentiation while suppressing Th2 and Th17 responses, potentially counteracting the inflammatory cascades characteristic of airway disease [85].

Beyond SCFAs, other microbial metabolites including tryptophan derivatives, bile acids, and lipopolysaccharides contribute to gut–lung axis signaling. Tryptophan metabolites produced by intestinal bacteria can activate the aryl hydrocarbon receptor (AhR), promoting IL-22 production and supporting epithelial barrier integrity in both the gut and lung [86]. Dysbiosis characterized by reduced tryptophan metabolism may therefore compromise respiratory barrier function and increase susceptibility to environmental triggers. While specific measurements of these metabolites in horses with severe asthma are lacking, the conserved nature of these pathways across mammalian species suggests their likely relevance in equine respiratory disease.

### 4.4. Alterations in Archaeal and Fungal Fecal Microbiota of Horses with SEA

Comparative studies in human asthma have demonstrated significant associations between gut microbiota composition and disease severity, with specific implications for fungal and archaeal communities in modulating inflammatory responses [87]. Studies evaluating the fecal archaeal composition of healthy adult horses have reported *Methanocorpusculum labreanum*, a methanogen belonging to the archaea genus Methanocorpusculum, as the most abundant methane-producing microorganism in the feces of thoroughbred horses [88]. A detailed metagenomic analysis in Przewalski’s horses following anthelmintic treatment provided insights into archaeal community dynamics and functional roles [89]. In this population, Methanobrevibacter emerged as the dominant archaeal genus within Euryarchaeota. Anthelmintic treatment significantly impacted archaeal communities, increasing Methanobrevibacter abundance while decreasing Methanocorpusculum, including the species *Methanocorpusculum labreanum* and *Methanocorpusculum bavaricum*. These alterations in methanogenic archaea composition have implications for fiber degradation efficiency, as methanogens enhance the activity of anaerobic fungi through interspecies hydrogen transfer [90]. In contrast, our study detected higher abundance of another methanogenic archaea genus, Metanimicrococcus, in the feces of healthy horses, while the archaea genus *Candidatus Nitrosocosmicus* (order Nitrososphaerales) was higher in the SEA group. Previously, higher relative abundances of Methanimicrococcus have been associated with methylotrophic methanogenesis, contributing to hydrogen balance in the hindgut of healthy horses [91]. Higher abundance of *Candidatus Nitrosocosmicus*, an ammonia-oxidizing archaeon, suggests altered nitrogen cycling, indicating functional changes in archaeal metabolism that may affect fermentation efficiency and microbial homeostasis [92]. Our findings suggest that SEA is associated with shifts in archaeal metabolic potential that could impact hindgut energy utilization and microbial ecology. The functional significance of methanogenic archaea extends beyond methane production. These microorganisms participate in complex syntrophic relationships with bacterial and fungal fermenters, removing metabolic end-products like hydrogen (H2) and formate (HCO2¯) that would otherwise inhibit fermentation [93]. The diversity and abundance of methanogens can influence overall hindgut fermentation efficiency and, consequently, the nutritional status and metabolic health of the host [94].

To the authors’ knowledge, there are no specific studies directly linking archaeal or fungal dysbiosis to severe equine asthma; however, the broader context of the gut–lung axis suggests that disruptions in archaeal/fungi communities could contribute to systemic inflammatory states through altered metabolite production and immune modulation. Anaerobic fungi represent a unique and potent component of the hindgut of herbivores species including horses, with specialized capabilities for plant cell wall degradation that complement bacterial fermentation [95]. Edwards et al. [48] demonstrated that anaerobic fungi were present in all Equidae (horses, donkeys, and zebra) fecal samples examined; however, no core fungal taxon was universally detected. The most abundant fungi genera identified were Caecomyces, Piromyces, and Neocallimastix, with significant variation in community composition related to equine type and individual animal characteristics in healthy animals [48]. In contrast, this present study revealed a more distinct fungi microbial profile in the feces of horses than previously reported by others [48,96]. For instance, fungi genera *Alternaria*, *Ascochyta*, and *Cladosporium* were higher in the healthy horses compared to the horses with severe asthma. Unlike the anaerobic fungi from the phylum Neocallimastigomycota (such as Piromyces and Neocallimastix) that are specialized hindgut commensals in herbivores [48], *Alternaria*, *Ascochyta*, and *Cladosporium* are generally transient passengers in the gastrointestinal tract, entering primarily through dietary ingestion [97]. In the present study, although fecal samples were collected from horses in different locations, all the horses were under similar feeding conditions (i.e., SEA horses were not receiving special feeding such as steamed hay; Table 1). Additionally, given the number of animals in the current study, we did not account for feeding types or geographic location. Therefore, conclusions on fungal microbial composition based on dietary regimes cannot be made. Alternatively, the presence of substantial environmental fungi in the feces of healthy horses may actually reflect optimal immune tolerance mechanisms. In severe asthma, gut–lung axis dysfunction disrupts this tolerant state [98]. Chronic pulmonary inflammation generates systemic inflammatory mediators that reach the gut through circulation, activating intestinal immune responses and potentially breaking oral tolerance mechanisms [99]. Studies demonstrating increased intestinal permeability in asthma models show that barrier dysfunction allows microbial components to translocate across the epithelium [100]. While this translocation contributes to systemic inflammation, it may simultaneously reduce the amount of intact fungal material remaining in the intestinal lumen to be excreted in feces. We speculate that enhanced phagocytic activity by intestinal macrophages and dendritic cells in the inflamed gut of SEA horses could capture and degrade fungal spores more efficiently than in healthy horses, reducing fecal recovery despite equivalent dietary intake. Overall, these mechanisms suggest that monitoring fecal environmental fungi could serve as both a temporal marker for management interventions and a potential window into gut–lung axis function and systemic immune status. Future studies should employ controlled feeding studies where healthy and severe asthmatic horses receive identical diets while monitoring fecal fungal abundance, inflammatory markers, and respiratory outcomes to reveal the underlying pathophysiological mechanisms.

The extent to which archaeal and fungal communities in the gut correlate with respiratory outcomes has received minimal attention in equine research. However, extrapolating from the known functions of these organisms suggests plausible mechanistic connections. Reduced methanogenic archaeal diversity could impair hindgut fermentation efficiency, potentially altering SCFA production profiles and consequently affecting systemic immune regulation [93]. Similarly, diminished anaerobic fungal populations might reduce fiber degradation capacity, limiting substrate availability for SCFA-producing bacteria and creating a cascade effect on metabolite-mediated immune modulation.

Overall, understanding the gut microbiota–respiratory axis in horses could lead to innovative therapeutic strategies, including fecal microbiota transplantation, probiotic supplementation, and prebiotic interventions specifically designed to improve asthma management. Targeted microbiota interventions represent a promising therapeutic strategy that warrants further investigation in equine asthma. For instance, based on our archaea and fungal microbiota findings, horses with severe neutrophilic asthma under certain dietary regimes that do not have access to good quality hay could benefit from the ingestion of steamed hay to help alleviate the gut dysbiosis, thereby reducing systemic inflammation in the host. Furthermore, the fact that horses in similar environments develop asthma of different severity with variable BALF neutrophil infiltration can be attributed to multifactorial biological factors, of which gut microbiota composition represents a substantial contributor.

### 4.5. Study Limitations

The translational implications of cross-species gut microbiota investigations for equine asthma are substantial. While horses possess unique dietary requirements and gut physiology as obligate herbivores and hindgut fermenters, fundamental immunological pathways in the gut–lung communication appear remarkably conserved across mammals [23]. However, before further conclusions can be drawn, some caveats must be made. In the present study, fecal samples were not evaluated for SCFA composition; thus our discussion was based on the published literature that has shown the correlation between microbial populations and SCFA production and its benefit to the host. Additionally, given our limited number of animals per group, the addition of demographic characteristics in the statistical model was not feasible. However, as described in Section 2, we accounted for the difference in experimental units per group in the statistical model by performing permutation analyses to detect changes in the microbiota profile between groups. We acknowledge that the degree of inter- and intra-individual variability in equine fecal microbiota, coupled with potential environmental effects, makes it difficult to identify disease-specific microbial signatures without very large sample sizes and careful experimental control. Establishing causal relationships between microbiota alterations and disease outcomes requires longitudinal studies with repeated sampling, intervention trials, and potentially gnotobiotic animal models. However, such approaches are logistically challenging and expensive in large animals like horses. Future studies should aim to induce exacerbation without altering the diet, for instance by maintaining horses in remission on high-quality hay and subsequently modulating antigen exposure through environmental adjustments, thereby allowing a clearer assessment of microbiota–asthma relationships.

## 5. Conclusions

The present study demonstrated relevant alterations in the fecal microbiota composition of healthy horses and horses with severe neutrophilic asthma. Microbial alterations included low abundance of *Fibrobacter*, *Christensenella* and *Blautia*, which are key bacterial species involved in metabolic health and fiber degradation, in SEA-affected horses. In addition to bacterial dysbiosis, horses with SEA had very low fecal abundance of *Alternaria*, *Ascochyta*, and *Cladosporium*. In particular, *Alternaria* and *Cladosporium* are fungal genera associated with allergic respiratory diseases across species; however, their low abundance in the feces of SEA horses may be attributed to the gut–lung axis dysfunction. The present study also reported that archaea genus *Candidatus Nitrosocosmicus* was higher in the horses with SEA. These findings suggest that SEA is associated with significant shifts in both archaeal composition and community structure, which impact hydrogen metabolism, methanogenesis, and fermentative energy extraction in the equine hindgut. In summary, equine asthma represents a heterogeneous syndrome with distinct phenotypes and varying disease trajectories, which aligns with our hypothesis that microbiota composition contributes substantially to SEA heterogeneity.

## Figures and Tables

**Table 1:** Demographic characteristics of horses enrolled in this study.

Animal *	Location	Age (Years)	Breed	Sex	Use	Concentrate Feed	Roughage
H1	Texas	8	Thoroughbred	Male	Trail riding, some polo	none	Coastal/Bermuda hay, Alfalfa hay
H2	Virginia	14	Quarter Horse	Female	English performance, trail riding	Oats (soaked)	Hay cubes, Orchard hay, Alfalfa Hay, Grass mix of orchard timothy, brome, Kentucky blue grass
H3	Virginia	8	Paint	Female	English performance, trail riding	Oats (soaked), Alfalfa pellets (soaked)	Hay cubes, Orchard hay, Alfalfa Hay, Grass mix of orchard timothy, brome, Kentucky blue grass
H4	Texas	7	Quarter Horse	Female	Backyard (no riding)	Senior feed/Complete feed	Coastal/Bermuda hay, Alfalfa hay
H5	Texas	5	Quarter Horse	Female	Western performance	Senior feed/Complete feed	Alfalfa Hay
H6	Texas	20	Quarter Horse	Female	Backyard (no riding)	Senior feed/Complete feed	Coastal/Bermuda hay, Alfalfa hay
H7	Texas	9	Quarter Horse	Male	Ranch and roping	Flax grain 14%	Johnson/Coastal (Stephenville)
H8	Texas	5	Quarter Horse	Male	Backyard (no riding)	Senior feed/Complete feed	Coastal/Bermuda hay, Alfalfa hay
S1	Virginia	12	Thoroughbred	Male	Eventing	Senior feed/Complete feed	Orchard hay, Alfalfa hay
S2	Texas	17	Thoroughbred	Female	Trail riding	Senior feed/Complete feed	Coastal/Bermuda hay, Alfalfa hay
S3	Hawaii	16	Mini	Male	Backyard (no riding), therapy horse	Senior feed/Complete feed (soaked)	Pasture grazing
S4	Hawaii	9	Quarter Horse	Male	Barrel racing, Western performance, Trail riding, competition	Senior feed/Complete feed	Hay Cubes, Soaked

* H = healthy horses; S = horses with severe equine asthma.

**Table 2:** Bronchoalveolar lavage fluid (BALF) cytology of each horse enrolled in this study.

Animal *	Neutrophils %	Eosinophils %	Mast Cells %
H1	1	1	1
H2	1	0	2
H3	1	0	1
H4	2	1	1
H5	1	1	1
H6	5	0	1
H7	1	1	1
H8	2	1	1
S1	27	0	2
S2	23	3	0
S3	26	0	1
S4	47	0	1

* H = healthy horses; S = horses with severe equine asthma.

**Table 3:** Primers used for amplification of bacteria, fungi, and archaea in fecal samples in this study.

Target	Primer	Sequence (5′-3′)
Bacteria	28-F	GAGTTTGATCNTGGCTCAG
	519-R	GTNTTACNGCGGCKGCTG
Fungi	ITS1-F	CTTGGTCATTTAGAGGAAGTAA
	ITS2a-R	GCTGCGTTCTTCATCGATGC
Archaea	Arch517-F	GCYTAAAGSRNCCGTAGC
	Arch909-R	TTTCAGYCTTGCGRCCGTAC

## Data Availability

The raw sequencing data generated in this study have been deposited in the NCBI Sequence Read Archive (SRA) under accession number PRJNA1400632 for BioProject. These data are publicly available and can be accessed through the SRA database at https://dataview.ncbi.nlm.nih.gov/object/PRJNA1400632?reviewer=5f3rovi6qtfloc9nbgs06a7b51, accessed on 15 February 2026.

## Supplementary Materials

The following supporting information can be downloaded at: https://www.mdpi.com/article/10.3390/microorganisms14020484/s1, Figure S1: Principal Coordinates Analysis based on unweighted Unifrac distances for distinct fungal taxa in fecal samples of healthy horses (n = 8) and horses with severe equine asthma (SEA; n = 4).

## Abbreviations

The following abbreviations are used in this manuscript:

BAL	Bronchoalveolar lavage
BALF	Bronchoalveolar lavage fluid
SEA	Severe equine asthma
OTUs	Operational taxonomic units
ZOTUs	Zero-radius operational taxonomic units

## References

[1] White S.J., Moore-Colyer M., Marti E., Hannant D., Gerber V., Coüetil L., Richard E.A., Alcocer M. (2019). Antigen array for serological diagnosis and novel allergen identification in severe equine asthma. Sci. Rep..

[2] Gerber V., Tessier C., Marti E. (2015). Genetics of upper and lower airway diseases in the horse. Equine Vet. J..

[3] Tessier L., Côté O., Clark M.E., Viel L., Diaz-Méndez A., Anders S., Bienzle D. (2017). Impaired response of the bronchial epithelium to inflammation characterizes severe equine asthma. BMC Genom..

[4] Meiseberg L.K., Delarocque J., de Buhr N., Ohnesorge B. (2024). Clinical variability of equine asthma phenotypes and analysis of diagnostic steps in phenotype differentiation. Acta Vet. Scand..

[5] Couetil L.L., Cardwell J.M., Gerber V., Lavoie J.P., Leguillette R., Richard E.A. (2016). Inflammatory Airway Disease of Horses–Revised Consensus Statement. J. Vet. Intern. Med..

[6] Bond S., Leguillette R., Richard E.A., Couetil L., Lavoie J.P., Martin J.G., Pirie R.S. (2018). Equine asthma: Integrative biologic relevance of a recently proposed nomenclature. J. Vet. Intern. Med..

[7] Begley L., Madapoosi S., Opron K., Ndum O., Baptist A., Rysso K., Erb-Downward J.R., Huang Y.J. (2018). Gut microbiota relationships to lung function and adult asthma phenotype: A pilot study. BMJ Open Respir. Res..

[8] McGorum B.C., Dixon P.M., Halliwell R.E. (1993). Phenotypic analysis of peripheral blood and bronchoalveolar lavage fluid lymphocytes in control and chronic obstructive pulmonary disease affected horses, before and after ‘natural (hay and straw) challenges’. Vet. Immunol. Immunopathol..

[9] Lavoie J.P., Maghni K., Desnoyers M., Taha R., Martin J.G., Hamid Q.A. (2001). Neutrophilic airway inflammation in horses with heaves is characterized by a Th2-type cytokine profile. Am. J. Respir. Crit. Care Med..

[10] Moran G., Folch H., Henriquez C., Ortloff A., Barria M. (2012). Reaginic antibodies from horses with recurrent airway obstruction produce mast cell stimulation. Vet. Res. Commun..

[11] Kleiber C., McGorum B.C., Horohov D.W., Pirie R.S., Zurbriggen A., Straub R. (2005). Cytokine profiles of peripheral blood and airway CD4 and CD8 T lymphocytes in horses with recurrent airway obstruction. Vet. Immunol. Immunopathol..

[12] Biesbroek G., Tsivtsivadze E., Sanders E.A., Montijn R., Veenhoven R.H., Keijser B.J., Bogaert D. (2014). Early respiratory microbiota composition determines bacterial succession patterns and respiratory health in children. Am. J. Respir. Crit. Care Med..

[13] Martinez F.D., Guerra S. (2018). Early Origins of Asthma. Role of Microbial Dysbiosis and Metabolic Dysfunction. Am. J. Respir. Crit. Care Med..

[14] Schwarzer M., Srutkova D., Schabussova I., Hudcovic T., Akgün J., Wiedermann U., Kozakova H. (2013). Neonatal colonization of germ-free mice with Bifidobacterium longum prevents allergic sensitization to major birch pollen allergen Bet v 1. Vaccine.

[15] Morin S., Fischer R., Przybylski-Nicaise L., Bernard H., Corthier G., Rabot S., Wal J.M., Hazebrouck S. (2012). Delayed bacterial colonization of the gut alters the host immune response to oral sensitization against cow’s milk proteins. Mol. Nutr. Food Res..

[16] Gensollen T., Blumberg R.S. (2017). Correlation between early-life regulation of the immune system by microbiota and allergy development. J. Allergy Clin. Immunol..

[17] Schank N., Cottone A., Wulf M., Seiter K., Thomas B., Miller L.M.J., Anderson S.L., Sahyoun A., Abidi A.H., Kassan M. (2025). The Role of Short-Chain Fatty Acids (SCFAs) in Colic and Anti-Inflammatory Pathways in Horses. Animals.

[18] Vargas A., Robinson B.L., Houston K., Vilela Sangay A.R., Saadeh M., D’Souza S., Johnson D.A. (2025). Gut microbiota-derived metabolites and chronic inflammatory diseases. Explor. Med..

[19] Marsland B.J., Trompette A., Gollwitzer E.S. (2015). The Gut–Lung Axis in Respiratory Disease. Ann. Am. Thorac. Soc..

[20] Enaud R., Prevel R., Ciarlo E., Beaufils F., Wieërs G., Guery B., Delhaes L. (2020). The Gut–Lung Axis in Health and Respiratory Diseases: A Place for Inter-Organ and Inter-Kingdom Crosstalks. Front. Cell. Infect. Microbiol..

[21] Frati F., Salvatori C., Incorvaia C., Bellucci A., Di Cara G., Marcucci F., Esposito S. (2018). The Role of the Microbiome in Asthma: The Gut–Lung Axis. Int. J. Mol. Sci..

[22] Sun M., Lu F., Yu D., Wang Y., Chen P., Liu S. (2024). Respiratory diseases and gut microbiota: Relevance, pathogenesis, and treatment. Front. Microbiol..

[23] Leduc L., Costa M., Leclère M. (2024). The Microbiota and Equine Asthma: An Integrative View of the Gut–Lung Axis. Animals.

[24] Kaiser-Thom S., Hilty M., Gerber V. (2020). Effects of hypersensitivity disorders and environmental factors on the equine intestinal microbiota. Vet. Q..

[25] Leclere M., Costa M.C. (2020). Fecal microbiota in horses with asthma. J. Vet. Intern. Med..

[26] Costa M.C., Silva G., Ramos R.V., Staempfli H.R., Arroyo L.G., Kim P., Weese J.S. (2015). Characterization and comparison of the bacterial microbiota in different gastrointestinal tract compartments in horses. Vet. J..

[27] Mazan M.R., Hoffman A.M. (2003). Clinical techniques for diagnosis of inflammatory airway disease in the horse. Clin. Tech. Equine Pract..

[28] Pickles K., Pirie R.S., Rhind S., Dixon P.M., McGorum B.C. (2002). Cytological analysis of equine bronchoalveolar lavage fluid. Part 2: Comparison of smear and cytocentrifuged preparations. Equine Vet. J..

[29] Davis K.U., Sheats M.K. (2019). Bronchoalveolar Lavage Cytology Characteristics and Seasonal Changes in a Herd of Pastured Teaching Horses. Front. Vet. Sci..

[30] Li M., Mao J., Diaz I., Kopylova E., Melnik A.V., Aksenov A.A., Tipton C.D., Soliman N., Morgan A.M., Boyd T. (2023). Multi-omic approach to decipher the impact of skincare products with pre/postbiotics on skin microbiome and metabolome. Front. Med..

[31] Hoffman C., Siddiqui N.Y., Fields I., Gregory W.T., Simon H.M., Mooney M.A., Wolfe A.J., Karstens L. (2021). Species-Level Resolution of Female Bladder Microbiota from 16S rRNA Amplicon Sequencing. mSystems.

[32] Allen H.K., Bayles D.O., Looft T., Trachsel J., Bass B.E., Alt D.P., Bearson S.M., Nicholson T., Casey T.A. (2016). Pipeline for amplifying and analyzing amplicons of the V1–V3 region of the 16S rRNA gene. BMC Res. Notes.

[33] Wimmer-Scherr C., Taminiau B., Renaud B., van Loon G., Palmers K., Votion D., Amory H., Daube G., Cesarini C. (2021). Comparison of Fecal Microbiota of Horses Suffering from Atypical Myopathy and Healthy Co-Grazers. Animals.

[34] Loublier C., Taminiau B., Heinen J., Lecoq L., Amory H., Daube G., Cesarini C. (2023). Evaluation of Bacterial Composition and Viability of Equine Feces after Processing for Transplantation. Microorganisms.

[35] Tipton C.D., Sanford N.E., Everett J.A., Gabrilska R.A., Wolcott R.D., Rumbaugh K.P., Phillips C.D. (2019). Chronic wound microbiome colonization on mouse model following cryogenic preservation. PLoS ONE.

[36] Edgar R.C. (2010). Search and clustering orders of magnitude faster than BLAST. Bioinformatics.

[37] Zhang J., Kobert K., Flouri T., Stamatakis A. (2014). PEAR: A fast and accurate Illumina Paired-End reAd mergeR. Bioinformatics.

[38] Edgar R.C. (2016). UNOISE2: Improved error-correction for Illumina 16S and ITS amplicon sequencing. bioRxiv.

[39] Edgar R.C. (2016). SINTAX: A simple non-Bayesian taxonomy classifier for 16S and ITS sequences. bioRxiv.

[40] Edgar R.C. (2004). MUSCLE: Multiple sequence alignment with high accuracy and high throughput. Nucleic Acids Res..

[41] Nawrocki E.P., Kolbe D.L., Eddy S.R. (2009). Infernal 1.0: Inference of RNA alignments. Bioinformatics.

[42] Price M.N., Dehal P.S., Arkin A.P. (2010). FastTree 2—Approximately Maximum-Likelihood Trees for Large Alignments. PLoS ONE.

[43] Dixon P. (2003). VEGAN, a package of R functions for community ecology. J. Veg. Sci..

[44] McMurdie P.J., Holmes S. (2013). phyloseq: An R Package for Reproducible Interactive Analysis and Graphics of Microbiome Census Data. PLoS ONE.

[45] Chao A., Chiu C.-H., Jost L. (2014). Unifying Species Diversity, Phylogenetic Diversity, Functional Diversity, and Related Similarity and Differentiation Measures Through Hill Numbers. Annu. Rev. Ecol. Evol. Syst..

[46] Lozupone C., Lladser M.E., Knights D., Stombaugh J., Knight R. (2011). UniFrac: An effective distance metric for microbial community comparison. Isme J.

[47] Wang Z., Yu J., Liu Y., Gong J., Hu Z., Liu Z. (2025). Role of the microbiota-gut-lung axis in the pathogenesis of pulmonary disease in children and novel therapeutic strategies. Front. Immunol..

[48] Edwards J.E., Shetty S.A., van den Berg P., Burden F., van Doorn D.A., Pellikaan W.F., Dijkstra J., Smidt H. (2020). Multi-kingdom characterization of the core equine fecal microbiota based on multiple equine (sub)species. Anim. Microbiome.

[49] Dougal K., de la Fuente G., Harris P.A., Girdwood S.E., Pinloche E., Geor R.J., Nielsen B.D., Schott H.C., Elzinga S., Newbold C.J. (2014). Characterisation of the faecal bacterial community in adult and elderly horses fed a high fibre, high oil or high starch diet using 454 pyrosequencing. PLoS ONE.

[50] Rios-Covian D., Salazar N., Gueimonde M., de Los Reyes-Gavilan C.G. (2017). Shaping the Metabolism of Intestinal Bacteroides Population through Diet to Improve Human Health. Front. Microbiol..

[51] Ericsson A.C., Johnson P.J., Lopes M.A., Perry S.C., Lanter H.R. (2016). A Microbiological Map of the Healthy Equine Gastrointestinal Tract. PLoS ONE.

[52] Julliand V., de Vaux A., Millet L., Fonty G. (1999). Identification of Ruminococcus flavefaciens as the predominant cellulolytic bacterial species of the equine cecum. Appl. Environ. Microbiol..

[53] Steelman S.M., Chowdhary B.P., Dowd S., Suchodolski J., Janečka J.E. (2012). Pyrosequencing of 16S rRNA genes in fecal samples reveals high diversity of hindgut microflora in horses and potential links to chronic laminitis. BMC Vet. Res..

[54] Dougal K., Harris P.A., Edwards A., Pachebat J.A., Blackmore T.M., Worgan H.J., Newbold C.J. (2012). A comparison of the microbiome and the metabolome of different regions of the equine hindgut. FEMS Microbiol. Ecol..

[55] Shepherd M.L., Swecker W.S., Jensen R.V., Ponder M.A. (2012). Characterization of the fecal bacteria communities of forage-fed horses by pyrosequencing of 16S rRNA V4 gene amplicons. FEMS Microbiol. Lett..

[56] Everard A., Belzer C., Geurts L., Ouwerkerk J.P., Druart C., Bindels L.B., Guiot Y., Derrien M., Muccioli G.G., Delzenne N.M. (2013). Cross-talk between Akkermansia muciniphila and intestinal epithelium controls diet-induced obesity. Proc. Natl. Acad. Sci. USA.

[57] O’Donnell M.M., Harris H.M.B., Ross R.P., O’Toole P.W. (2017). Core fecal microbiota of domesticated herbivorous ruminant, hindgut fermenters, and monogastric animals. Microbiologyopen.

[58] O’Donnell M.M., Harris H.M., Jeffery I.B., Claesson M.J., Younge B., O’Toole P.W., Ross R.P. (2013). The core faecal bacterial microbiome of Irish Thoroughbred racehorses. Lett. Appl. Microbiol..

[59] Park T., Cheong H., Yoon J., Kim A., Yun Y., Unno T. (2021). Comparison of the Fecal Microbiota of Horses with Intestinal Disease and Their Healthy Counterparts. Vet. Sci..

[60] Lara F., Castro R., Thomson P. (2022). Changes in the gut microbiome and colic in horses: Are they causes or consequences?. Open Vet. J..

[61] Durack J., Boushey H.A., Lynch S.V. (2016). Airway Microbiota and the Implications of Dysbiosis in Asthma. Curr. Allergy Asthma Rep..

[62] Hufnagl K., Pali-Schöll I., Roth-Walter F., Jensen-Jarolim E. (2020). Dysbiosis of the gut and lung microbiome has a role in asthma. Semin. Immunopathol..

[63] Liang W., Yang Y., Gong S., Wei M., Ma Y., Feng R., Gao J., Liu X., Tu F., Ma W. (2023). Airway dysbiosis accelerates lung function decline in chronic obstructive pulmonary disease. Cell Host Microbe.

[64] Pu Q., Lin P., Gao P., Wang Z., Guo K., Qin S., Zhou C., Wang B., Wu E., Khan N. (2021). Gut Microbiota Regulate Gut–Lung Axis Inflammatory Responses by Mediating ILC2 Compartmental Migration. J. Immunol..

[65] Fillion-Bertrand G., Dickson R.P., Boivin R., Lavoie J.P., Huffnagle G.B., Leclere M. (2019). Lung Microbiome Is Influenced by the Environment and Asthmatic Status in an Equine Model of Asthma. Am. J. Respir. Cell Mol. Biol..

[66] McKinney C.A., Oliveira B.C.M., Bedenice D., Paradis M.R., Mazan M., Sage S., Sanchez A., Widmer G. (2020). The fecal microbiota of healthy donor horses and geriatric recipients undergoing fecal microbial transplantation for the treatment of diarrhea. PLoS ONE.

[67] Costa M.C., Weese J.S. (2012). The equine intestinal microbiome. Anim. Health Res. Rev..

[68] Radcliffe R.M. (2016). Anaphylaxis. Equine Clinical Immunology.

[69] Liu J., Hong W., Sun Z., Zhang S., Xue C., Dong N. (2025). The gut–lung axis: Effects and mechanisms of gut microbiota on pulmonary diseases. Front. Immunol..

[70] Liu Y., Dai J., Zhou G., Chen R., Bai C., Shi F. (2025). Innovative Therapeutic Strategies for Asthma: The Role of Gut Microbiome in Airway Immunity. J. Asthma Allergy.

[71] Tang D., Wang C., Liu H., Wu J., Tan L., Liu S., Lv H., Wang C., Wang F., Liu J. (2024). Integrated Multi-Omics Analysis Reveals Mountain-Cultivated Ginseng Ameliorates Cold-Stimulated Steroid-Resistant Asthma by Regulating Interactions among Microbiota, Genes, and Metabolites. Int. J. Mol. Sci..

[72] Ray A., Kolls J.K. (2017). Neutrophilic Inflammation in Asthma and Association with Disease Severity. Trends Immunol..

[73] Ouyang W., Kolls J.K., Zheng Y. (2008). The Biological Functions of T Helper 17 Cell Effector Cytokines in Inflammation. Immunity.

[74] Kao C.Y., Chen Y., Thai P., Wachi S., Huang F., Kim C., Harper R.W., Wu R. (2004). IL-17 markedly up-regulates beta-defensin-2 expression in human airway epithelium via JAK and NF-kappaB signaling pathways. J. Immunol..

[75] Sorbello V., Ciprandi G., Di Stefano A., Massaglia G.M., Favatà G., Conticello S., Malerba M., Folkerts G., Profita M., Rolla G. (2015). Nasal IL-17F is related to bronchial IL-17F/neutrophilia and exacerbations in stable atopic severe asthma. Allergy.

[76] Ricciardolo F.L.M., Sorbello V., Folino A., Gallo F., Massaglia G.M., Favatà G., Conticello S., Vallese D., Gani F., Malerba M. (2017). Identification of IL-17F/frequent exacerbator endotype in asthma. J. Allergy Clin. Immunol..

[77] Chambers E.S., Nanzer A.M., Pfeffer P.E., Richards D.F., Timms P.M., Martineau A.R., Griffiths C.J., Corrigan C.J., Hawrylowicz C.M. (2015). Distinct endotypes of steroid-resistant asthma characterized by IL-17A(high) and IFN-γ(high) immunophenotypes: Potential benefits of calcitriol. J. Allergy Clin. Immunol..

[78] Zhang M., Qin Z., Huang C., Liang B., Zhang X., Sun W. (2025). The gut microbiota modulates airway inflammation in allergic asthma through the gut–lung axis related immune modulation: A review. Biomol. Biomed..

[79] Wilson N.G., Hernandez-Leyva A., Schwartz D.J., Bacharier L.B., Kau A.L. (2024). The gut metagenome harbors metabolic and antibiotic resistance signatures of moderate-to-severe asthma. FEMS Microbes.

[80] Wang Q., Ji J., Xiao S., Wang J., Yan X., Fang L. (2025). Explore Alteration of Lung and Gut Microbiota in a Murine Model of OVA-Induced Asthma Treated by CpG Oligodeoxynucleotides. J. Inflamm. Res..

[81] Lv J., Zhang Y., Liu S., Wang R., Zhao J. (2025). Gut–lung axis in allergic asthma: Microbiota-driven immune dysregulation and therapeutic strategies. Front. Pharmacol..

[82] Deng X., Wu X., Wang R., Qiao X., Cao T., Xu Y., Jin Q., Jia L., Liang W. (2025). Gut microbiota-based biomarkers for precision subtype classification and mechanistic understanding of biliary and hyperlipidemic acute pancreatitis. Front. Microbiol..

[83] Muralitharan R.R., Zheng T., Dinakis E., Xie L., Barbaro-Wahl A., Jama H.A., Nakai M., Paterson M., Leung K.C., McArdle Z. (2025). Gut Microbiota Metabolites Sensed by Host GPR41/43 Protect Against Hypertension. Circ. Res..

[84] Liu X.F., Shao J.H., Liao Y.T., Wang L.N., Jia Y., Dong P.J., Liu Z.Z., He D.D., Li C., Zhang X. (2023). Regulation of short-chain fatty acids in the immune system. Front. Immunol..

[85] Zhang J., Zou Y., Chen L., Xu Q., Wang Y., Xie M., Liu X., Zhao J., Wang C.Y. (2022). Regulatory T Cells, a Viable Target Against Airway Allergic Inflammatory Responses in Asthma. Front. Immunol..

[86] Kang H., Chen Z., Wang B., Chen Z. (2025). The AhR/IL-22 axis in chronic gut inflammation: Unraveling mechanisms and therapeutic prospects. Front. Immunol..

[87] Kim Y.C., Sohn K.H., Kang H.R. (2024). Gut microbiota dysbiosis and its impact on asthma and other lung diseases: Potential therapeutic approaches. Korean J. Intern. Med..

[88] Lwin K.O., Matsui H. (2014). Comparative analysis of the methanogen diversity in horse and pony by using mcrA gene and archaeal 16s rRNA gene clone libraries. Archaea.

[89] Hu D., Yang J., Qi Y., Li B., Li K., Mok K.M. (2021). Metagenomic Analysis of Fecal Archaea, Bacteria, Eukaryota, and Virus in Przewalski’s Horses Following Anthelmintic Treatment. Front. Vet. Sci..

[90] Li Y., Meng Z., Xu Y., Shi Q., Ma Y., Aung M., Cheng Y., Zhu W. (2021). Interactions between Anaerobic Fungi and Methanogens in the Rumen and Their Biotechnological Potential in Biogas Production from Lignocellulosic Materials. Microorganisms.

[91] Murru F., Fliegerova K., Mura E., Mrázek J., Kopečný J., Moniello G. (2018). A comparison of methanogens of different regions of the equine hindgut. Anaerobe.

[92] Han S., Kim S., Sedlacek C.J., Farooq A., Song C., Lee S., Liu S., Brüggemann N., Rohe L., Kwon M. (2024). Adaptive traits of Nitrosocosmicus clade ammonia-oxidizing archaea. mBio.

[93] Volmer J.G., McRae H., Morrison M. (2023). The evolving role of methanogenic archaea in mammalian microbiomes. Front. Microbiol..

[94] Thomas C.M., Desmond-Le Quéméner E., Gribaldo S., Borrel G. (2022). Factors shaping the abundance and diversity of the gut archaeome across the animal kingdom. Nat. Commun..

[95] Hess M., Paul S.S., Puniya A.K., van der Giezen M., Shaw C., Edwards J.E., Fliegerová K. (2020). Anaerobic Fungi: Past, Present, and Future. Front. Microbiol..

[96] Edwards J.E., Schennink A., Burden F., Long S., van Doorn D.A., Pellikaan W.F., Dijkstra J., Saccenti E., Smidt H. (2020). Domesticated equine species and their derived hybrids differ in their fecal microbiota. Anim. Microbiome.

[97] Hallen-Adams H.E., Suhr M.J. (2017). Fungi in the healthy human gastrointestinal tract. Virulence.

[98] Zheng T., Huang Y., Yao H. (2025). Advances in the Gut–Lung Axis and Bronchial Asthma: From Mechanisms to Therapeutic Potential. Clin. Transl. Allergy.

[99] Zhao M.a., Chu J., Feng S., Guo C., Xue B., He K., Li L. (2023). Immunological mechanisms of inflammatory diseases caused by gut microbiota dysbiosis: A review. Biomed. Pharmacother..

[100] Di Vincenzo F., Del Gaudio A., Petito V., Lopetuso L.R., Scaldaferri F. (2024). Gut microbiota, intestinal permeability, and systemic inflammation: A narrative review. Intern. Emerg. Med..

