# Supplementary material for: Multi-Kingdom Fecal Microbiota Alterations in Horses with Severe Equine Asthma
Source: Microorganisms. 2026 Feb 17;14(2):484. doi: 10.3390/microorganisms14020484 (PMC12943238; doi:10.3390/microorganisms14020484)
Supplement: Supplementary file 1 [file microorganisms-14-00484-s001.zip › microorganisms-4154394-supplementary.pdf]

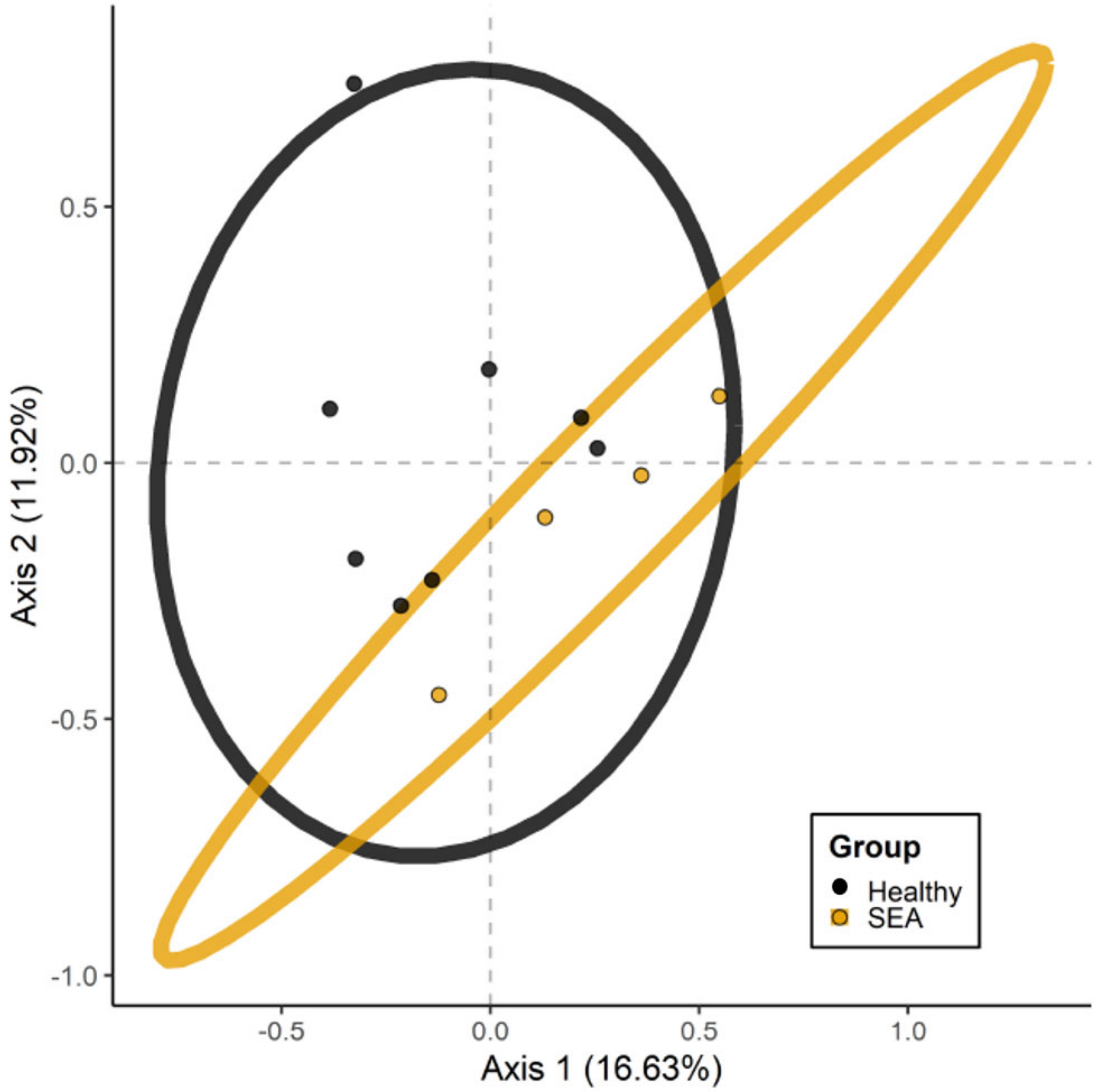

Figure S1. Principal Coordinates Analysis based on unweighted Unifrac distances for distinct fungal taxa in fecal samples of healthy horses (n = 8) and horses with severe equine asthma (SEA; n = 4).
